# Neuroinflammatory history results in overlapping transcriptional signatures with heroin exposure in the nucleus accumbens and alters responsiveness to heroin in male rats

**DOI:** 10.1038/s41398-024-03203-4

**Published:** 2024-12-19

**Authors:** Gabriele Floris, Mary Tresa Zanda, Konrad R. Dabrowski, Stephanie E. Daws

**Affiliations:** 1 Center for Substance Abuse Research, Temple University, Philadelphia, PA USA; 2 Department of Neural Sciences, Temple University, Philadelphia, PA USA; 3 Department of Biology, Temple University, Philadelphia, PA USA

**Keywords:** Molecular neuroscience, Addiction

## Abstract

Recent progress in psychiatric research has highlighted neuroinflammation in the pathophysiology of opioid use disorder (OUD), suggesting that heightened immune responses in the brain may exacerbate opioid-related mechanisms. However, the molecular mechanisms resulting from neuroinflammation that impact opioid-induced behaviors and transcriptional pathways remain poorly understood. In this study, we have begun to address this critical knowledge gap by exploring the intersection between neuroinflammation and exposure to the opioid heroin, utilizing lipopolysaccharide (LPS)-induced neuroinflammation, to investigate transcriptional changes in the nucleus accumbens (NAc), an essential region in the mesolimbic dopamine system that mediates opioid reward. By integrating RNA sequencing with bioinformatic and statistical analyses, we observed significant transcriptional overlaps between neuroinflammation and experimenter-administered heroin exposure in the NAc. Furthermore, we identified a subset of NAc genes synergistically regulated by LPS and heroin, suggesting that LPS history may exacerbate some heroin-induced molecular neuroadaptations. We extended our findings to examine the impact of neuroinflammatory history on responsiveness to heroin in a locomotor sensitization assay and observed LPS-induced exacerbation of heroin sensitization, indicating that neuroinflammation may increase sensitivity to opioids’ behavioral effects. Lastly, we performed comparative analysis of the NAc transcriptional profiles of LPS-heroin rats with those obtained from voluntary heroin intake in a rat model of heroin self-administration (SA) and published human OUD datasets. We observed significant convergence of the three datasets and identified transcriptional patterns in the preclinical models that recapitulated human OUD neuropathology, highlighting the utility of preclinical models to further investigate molecular mechanisms of OUD pathology. Overall, our study elucidates transcriptional interconnections between neuroinflammation and heroin exposure, and also provides evidence of the behavioral ramifications of such interactions. By bridging the gap between neuroinflammation and heroin exposure at the transcriptional level, our work provides valuable insights for future research aimed at mitigating the influence of inflammatory pathways in OUD.

## Introduction

Current psychiatric research suggests that neuroinflammation, a process characterized by heightened immune responses in the brain, may play a causal role in the pathophysiology of various psychiatric conditions [1]. Particularly, in opioid use disorder (OUD), neuroinflammation is believed to exacerbate the mechanisms of addiction, influencing drug-seeking behaviors and withdrawal symptoms [2]. Clinical evidence associating inflammation with OUD mainly stems from observations of heightened levels of proinflammatory cytokines in the bloodstream of individuals dependent on opioids [3], though studies on cytokine release in human cells report inconsistent outcomes [2]. Nevertheless, recent transcriptomic analyses in human brain samples from patients with OUD have indicated a clear link between dysregulation of the brain’s inflammatory pathways and opioid dependence [4–6]. Despite these advancements, our understanding of the specific transcriptional and molecular mechanisms by which opioids induce pro-inflammatory responses in the brain, and how these inflammatory processes, in turn, may affect heroin’s impact, remains limited. This gap highlights the need for further investigation into the intricate and specific interactions between neuroinflammation and opioid dependence, emphasizing the complexity of this bidirectional relationship. Therefore, investigation into the transcriptional changes at the intersection between neuroinflammation and heroin exposure may aid in the identification of previously understudied pathways that may contribute to drug seeking behavior or vulnerability for addiction-like phenotypes. The use of immunostimulant compounds such as lipopolysaccharide (LPS) has been employed to model phenotypes of depression and schizophrenia in rodents following systemic inflammation [7–10]. Emerging research in this domain has revealed that such inflammation may also impact the neurobiological response to drugs of abuse [11–15]. Systemic inflammation from peripheral LPS administration can potentiate or enhance the physiological and behavioral responses to nicotine, psychostimulants, and alcohol [16–18]. In our study, we have begun to further investigate this later theory by employing a rodent model of LPS-induced neuroinflammation [19, 20] to evaluate the transcriptional changes in the nucleus accumbens (NAc), a brain region pivotal in OUD and sensitive to immunomodulation [21]. This study employs a combination of RNA sequencing (seq) and bioinformatic analyses to investigate the impact of LPS and/or heroin on the NAc transcriptional landscape. The findings reveal a significant concordance in the transcriptional responses between pretreatment of LPS and heroin exposure in rats, with a subset of genes recapitulating human OUD neuropathologies. Moreover, LPS pretreatment amplified heroin-induced locomotor sensitization, suggesting that neuroinflammation may increase sensitivity to opioids. This research provides valuable insights for future research aimed at mitigating the influence of inflammatory pathways in OUD.

## Methods

### Subjects

Adult male Sprague Dawley rats (Charles River Laboratories), 8 weeks old, were used in this study. Rats were pair-housed upon arrival with constant room temperature (22 ± 2 °C) and humidity (40%). Rats were acclimated for 7 days prior to behavioral experiments. Rats used for non-SA studies were paired-housed upon arrival and kept on a normal light/dark cycle (lights on at 9:00 a.m.; off at 9:00 p.m.). SA rats were kept on a reverse light/dark cycle (lights on at 9:00 p.m.; off at 9:00 a.m.) and single-housed after intravenous catheter surgery for the remainder of the experiment, as previously described [22]. Rodent chow food was provided *ad libitum*, except where described. All procedures were compliant with the National Institutes of Health’s Guide for the Care and Use of Laboratory Animals and were approved by Temple University’s Institutional Animal Care and Use Committee.

### Drugs

Diamorphine hydrochloride (heroin) was provided by the NIDA Drug Supply, (Research Triangle Park, NC) and dissolved in a sterile saline solution (0.9% sodium chloride) for injection and intravenous SA. Lipopolysaccharides (LPS) from Escherichia coli was purchased from Sigma-Aldrich (St. Louis, MO) (catalog # L2630) and dissolved in a sterile saline solution for intraperitoneal injection.

### Heroin-induced locomotor sensitization

250 µg/kg LPS or saline was administered intraperitoneally (IP) every third day for a total number of five injections over a two-week period on days 1, 3, 6, 9, and 12 (Fig. S1A), as described in published studies [19, 20]. One week after the last LPS or saline injection, all animals began the heroin-induced locomotor sensitization procedure described in the subsequent section. To assess heroin-induced locomotor sensitization, we refined a rat model based on previous methodologies [23–25]. This model involved administering intermittent doses of heroin (0.5 mg/kg subcutaneously) or saline to rats on alternate days over a 5-day period, with locomotor activity for each treatment session recorded. To ensure accurate baseline measurements, rats’ locomotor activity was monitored for an hour before administering heroin, allowing them to acclimate to the environment. This was followed by a two-hour observation period post-heroin administration to capture any changes in activity. At the end of the intermittent heroin treatment, rats underwent a drug free period for 1 week, in their home-cage. This period aimed to simulate a drug-free interval before the subsequent assessment phase [23]. Following this interval, the intensity of locomotor sensitization was evaluated. The assessment commenced with an hour-long acclimation in the activity chamber to establish a baseline, after which a single challenge dose of heroin (0.5 mg/kg subcutaneously) was administered. The rats’ locomotor activity was then monitored for an additional two hours to quantify the effects of sensitization. The assessment of locomotor activity was performed as previously described [26]. Briefly, rats were individually placed into transparent plastic chambers (45 cm × 20 cm × 20 cm) that were set within metal frames containing 16 infrared light emitters and detectors. The intervening space between the beams was 2.5 cm and beam height were 4.5 cm. A computer interface with a dedicated software (Digiscan DMicro system, Accuscan, Inc., Columbus, OH) recorded the number of photocell beam breaks. The experiment was performed in two separate cohorts of rats on subsequent days. A total of 8 rats for each treatment group were used.

### Behavioral manipulations

For the RNA-seq study, 250 µg/kg LPS or saline was administered every third day, as described above, on days 1, 5, 8, 11 and 14, with modification to account for weekends (Fig. S1B). Four days later, animals received escalating doses of heroin or saline vehicle, IP, for five days: day 18- 1 mg/kg; day 19- 2 mg/kg; day 20- 3 mg/kg; day 21- 4 mg/kg; day 22- 5 mg/kg. The dose of heroin used in this portion of the study corresponded to the typical range of heroin SA we observe in adult male rats over 10 days (10–70 infusions). Fifteen minutes after the last injection on day 22, animals were placed into a locomotor activity chamber to record locomotion for 30 minutes, as described above. This brief exposure to the locomotor activity chamber was conducted to evaluate the effectiveness of the pharmacological treatment and to evaluate individual responses among all animals, with every subject undergoing this procedure. Animals were euthanized within one hour after the conclusion of the locomotor assay and brains removed for RNA-seq. The sample sizes were as follows: Saline-Saline, 8; LPS-saline, 8; Saline-Heroin, 6; LPS-Heroin, 8. The experiment was performed once in our laboratory. For rats that underwent heroin SA, procedures were performed in operant chambers (29.5 × 32.5 × 23.5 cm, Med Associates, Fairfax, VT, USA) under a fixed ratio (FR) 1 schedule, as previously described [22], at an infusion dose of 0.075 mg/kg heroin. Animals were trained to self-administer heroin for 10 days in 6 h (hr) daily sessions. 6 rats were used in each heroin and saline SA group. Immediately after the last heroin SA session, rats were euthanized, and brains removed for RNA-seq. The experiments described in this section were performed once in our laboratory.

### RNA extraction and RNA-seq

Total RNA was isolated from NAc (core and shell) tissue using the MIRvana Paris Protein & RNA Isolation Kit (Thermo Fisher Scientific, Waltham, MA, USA) according to manufacturer’s instructions, as previously described [27]. Total RNA was suspended in RNase free water and concentration was evaluated with a Qubit 3.0 Fluorometer using the Qubit RNA High Sensitivity Assay buffer (Invitrogen, Carlsbad, California, USA). RNA-seq analysis of mRNA expression was performed on 300ng of NAc total RNA from biological replicate, unpooled samples. Six samples were sequenced per treatment group. The mean RNA integrity (RIN) value of sequenced samples was 8.7 and samples were of high quality, as determined by Bioanalyzer analysis. Raw data can be accessed on the Gene Expression Omnibus repository (GEO # GSE198807). See supplemental information for full methodological details.

### Statistics

Data distribution and variance were assessed with Kolmogorov–Smirnov and Bartlett’s tests. Parametric data analyses were conducted using one-way ANOVA or two-way ANOVA as required. For subsequent Post hoc comparisons, Tukey’s test was utilized to correct for multiple comparisons, with significance levels determined at 0.05. Two-way Repeated Measures ANOVA was used to compare locomotor responses to heroin or saline across the first five days of the behavioral sensitization assay. A two-way ANOVA was used to compare behavioral responses on the challenge day. Statistical evaluations were conducted utilizing GraphPad software (Prism version 10; GraphPad, San Diego, CA), with error bars indicating the mean plus or minus the standard error of the mean (SEM). The ROUT method, implemented in GraphPad, was employed for outlier analysis. Fisher’s exact tests were used to determine overlap of transcriptional datasets using the GeneOverlap package in R Bioconductor, version 1.36.0. Rats were randomly assigned to experimental groups. Blinding was not performed during collection of data. Sample sizes were estimated based on similar studies in the neuroscience field.

## Results

### Gene ontology and pathway analysis reveal the intertwined dynamics of inflammation and heroin exposure in the NAc

**Fig. 1:**
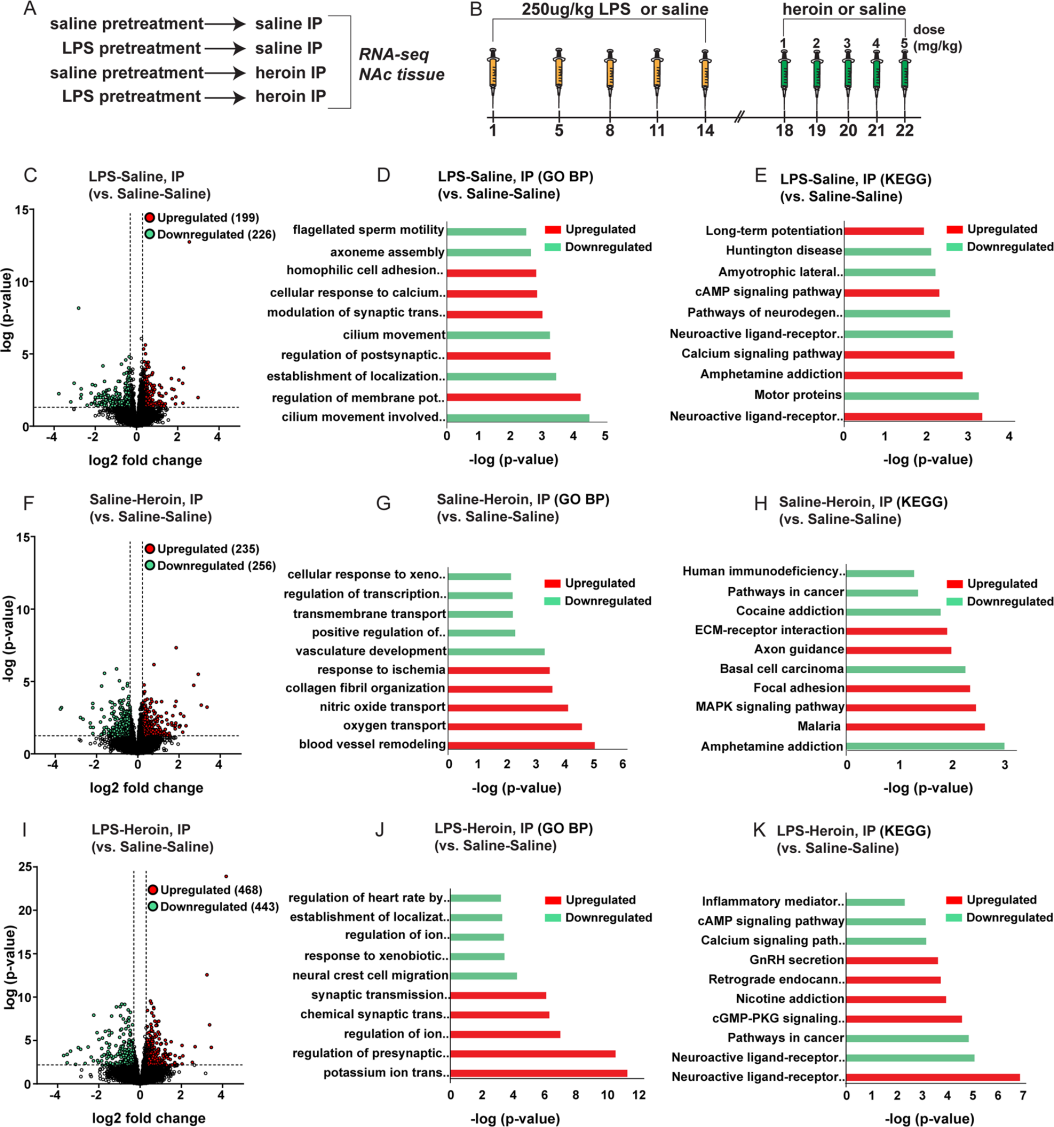
Subchronic LPS and heroin exposure regulate the NAc transcriptome in male rats. **A** Experimental design of RNA-seq study in the NAc tissue with experimental groups. **B** Schematic representation of experimental timeline. Rats received LPS or saline every third day. Four days later, rats received injection of heroin or saline for five consecutive days. Volcano plots (**C**, **F**, **I**) depicting differentially expressed genes (DEGs) in the NAc and bar graphs depicting Gene Ontology (**D**, **G**, **J**) or KEGG pathways analysis (**E**, **H**, **K**) of significantly altered pathways between Saline-Saline and: LPS-Saline (**C**, **D**, **E**), Saline-Heroin (**F**, **G**, **H**) or LPS-Heroin treated rats (**I**, **J**, **K**). Upregulated DEGs and pathways are represented in red, downregulated DEGs and pathways are represented in green.

To elucidate the transcriptomic signatures of neuroinflammation and heroin exposure, we used an unbiased RNA-seq approach, complemented by subsequent Gene Ontology (GO), Biological Processes (BP) and Kyoto Encyclopedia of Genes and Genomes (KEGG) pathway analysis. Our aim was to pinpoint critical pathways that may be commonly modulated by both LPS-induced neuroinflammation and heroin treatments, and furthermore synergistically heightened by the combined treatments. For this analysis, we included rats that experienced immune system activation with or without experiment-administered heroin (Fig. 1A). For experimenter-administered compounds, adult male Sprague Dawley rats were administered either saline or 250 µg/kg of the immunostimulant LPS intermittently over a period of two weeks. Subsequently, each animal group was given IP injections of either heroin or saline for a span of 5 days (Fig. 1B). The dose of heroin administered was increased each day from 1 to 5 mg/kg to mirror escalation of drug intake. We sought to investigate the enduring effects of immune stimulation on heroin-induced transcriptional changes and behavioral responses. While the acute administration of immune stimulants like LPS is known to influence neuroinflammatory markers, our objective extended beyond this to elucidate the residual impact of such stimulation on subsequent heroin-induced neuroadaptations. To this end, we timed the interval between LPS and heroin injections to isolate the lasting effects of LPS from any immediate ‘sickness behavior’ typically associated with its administration [28]. Our protocol for repeated LPS injections (5 injections, every other day), based on established research, utilizes lower-dose injections specifically designed to model chronic, medium-grade inflammation [19]. This approach is recognized for its relevance in major psychiatric conditions and its capacity to induce long-lasting alterations in both behavior and neural plasticity [19, 20, 29–31]. Supported by evidence of its significant long-lasting effects on behavior and underlying neural mechanisms, we hypothesized that this model might be an important tool for understanding how changes related to neural plasticity can alter brain sensitivity to drugs of abuse such as opioids. Moreover, this protocol produces a significant upregulation of neuroinflammatory markers such as interleukin-6 (IL-6), interleukin 1 beta (IL-1β), and tumor necrosis factor alpha (TNF-α) within 4 h of administration of LPS (250ug/kg) [19, 20, 32], all of which are relevant in the context of substance use disorders according to a recent study [33].

Following the final heroin or saline injection on day (D) 22, a brief assessment of locomotor activity (30 min) was monitored to evaluate the outcome of the treatments. A one-way ANOVA revealed a significant main effect of treatment (F(3,26) = 4.425, *p* = 0.0122) (Fig. S2). Upon the conclusion of behavioral assessments, animals were euthanized and tissue from the NAc was collected for RNA-seq analysis. Using this unbiased approach, we found that animals subjected to intermittent LPS immune-challenges (LPS-Saline) for 5 days exhibited a high number of differentially expressed genes (DEGs) compared to Saline-Saline controls, with a total of 425 DEGs, of which 199 were upregulated and 226 were downregulated (Fig. 1C and Table SE1). For the LPS-Saline group, the GO terms were primarily centered around synaptic processes (Fig. 1D). This includes regulation of membrane potential and synaptic transmission, indicative of an involvement in neuronal signaling and possibly synaptic plasticity. The KEGG pathway analysis complements this by highlighting pathways such as long-term potentiation and calcium signaling pathways, suggesting a focus on synaptic strength (Fig. 1E). Moreover, the neuroactive ligand-receptor interaction, a pathway associated with opioid addiction (Li et al., 2008), and pathways directly related to addiction, such as “amphetamine addiction,” were included following LPS alone, compared to the Saline-Saline group (Fig. 1E). In rats treated with intermittent heroin injections only, we observed 491 DEGs, with 235 upregulated and 256 downregulated compared to the Saline-Saline group (Fig. 1F and Table SE2). The Saline-Heroin IP group’s GO-BP terms shift towards vascular and tissue responses, with terms such as “blood vessel remodeling” and “response to ischemia” likely reflecting physiological adaptations to drug exposure (Fig. 1G). The KEGG pathways reveal the presence of “amphetamine addiction,” also present in the LPS-Saline group, along with “cocaine addiction,” compared to the Saline-Saline group (Fig. 1H). The highest number of DEGs were observed in animals receiving the combination of both LPS and heroin (911), with 468 upregulated and 443 downregulated, versus the Saline-Saline control group (Fig. 1I and Table SE3). Finally, the LPS-Heroin IP group presents an interesting blend of the terms observed in LPS alone or heroin alone groups. GO-BP terms relative to ion transmembrane transport as well as to synaptic processes such as synaptic transmission, glutamatergic synaptic transmission and calcium signaling pathways highlight similarity with the LPS group (Fig. 1J). On the other hand, GO-BP terms such as response to xenobiotic stimulus and regulation of heart rate by cardiac conduction are more indicative of the physiological and systemic responses seen in the Saline-Heroin group (Fig. 1J). Interestingly, we found in the KEGG pathways a repeated emphasis for the “neuroactive ligand-receptor interaction” pathway for both up- and down-regulated DEGs in the LPS-Heroin group, suggesting a complex regulation of the neurotransmitter systems (Fig. 1K). Moreover, “inflammatory mediator regulation of TRP channels” and pathways like “nicotine addiction,” elucidate the comprehensive responses elicited by concurrent LPS and heroin, relative to the Saline-Saline control group (Fig. 1K). Together, these data suggest that heroin exposure in rats with a history of LPS results in a substantially larger regulation of the NAc transcriptome.

Lastly, we compared the transcriptomes of rats pre-treated with LPS followed by heroin administration (LPS-Heroin) to those treated with either Saline-Heroin or LPS-Saline (Fig. S3 and Tables SE4–SE5). The primary aim was to isolate the specific impact of LPS pre-treatment on the biological response to heroin. This analysis includes an additional control to assess the baseline effects of inflammation alone, by comparing LPS-Heroin and LPS-Saline groups. Our comparative analyses elucidate differential gene expression patterns and pathway engagements, offering further insights into how prior inflammation may potentiate the effects of heroin. This approach also ensures a better control for potential confounding effects that may arise while comparing groups that differ by more than one variable. The GO and KEGG pathways for these comparisons can be found in Fig. S3D, E, G, and H.

### Significant overlap of NAc transcriptional signature between inflammation and heroin exposure

**Fig. 2:**
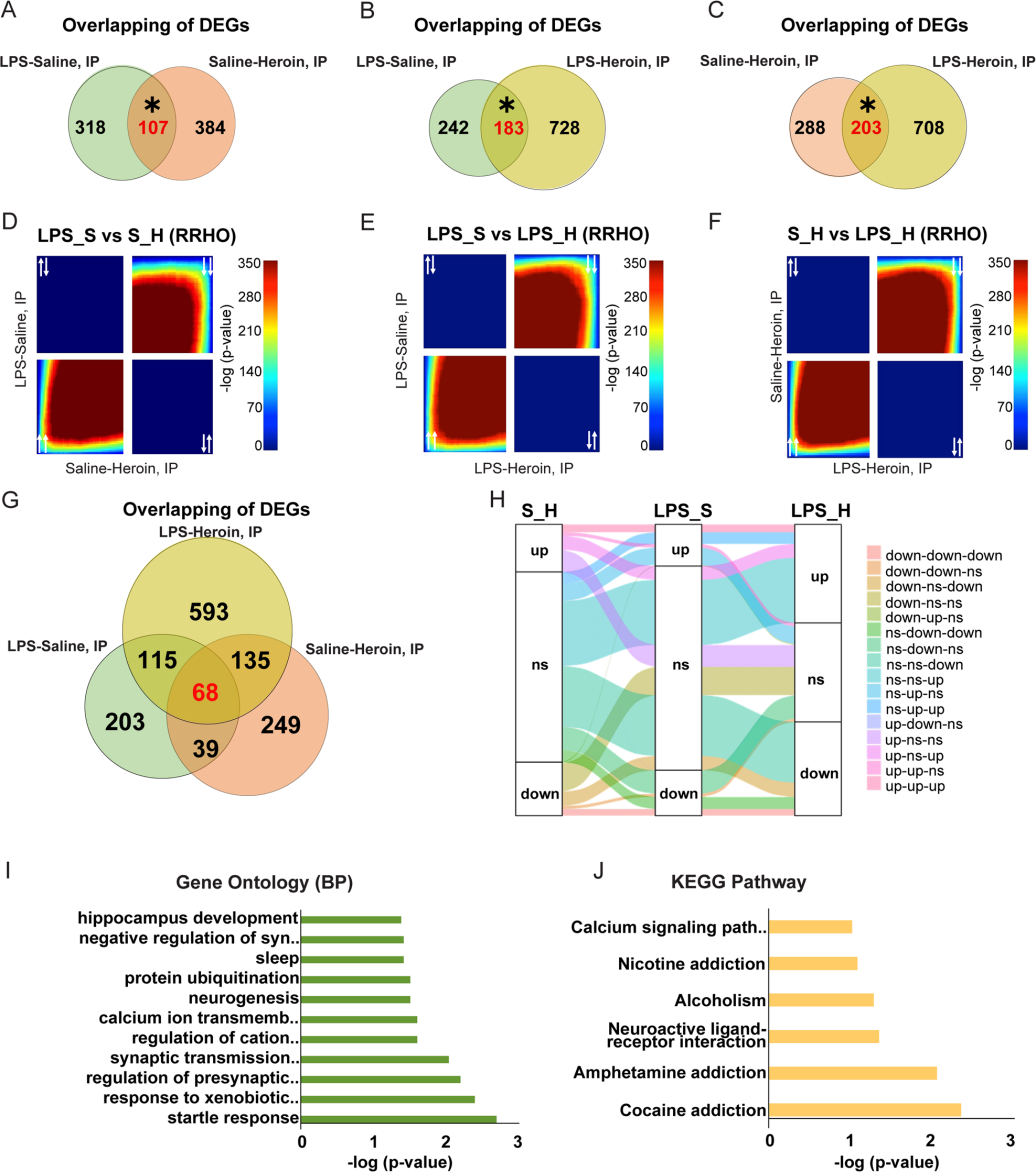
LPS and heroin exposure synergistically alter the NAc transcriptome. Venn diagram of total differential expressed genes (DEGs) regulated in the NAc by LPS-Saline and/or Saline-Heroin (**A**); LPS-Saline and/or LPS-Heroin (**B**); and Saline-Heroin and/or LPS-Heroin (**C**). In the intersection, in red, are represented the genes co-regulated in both groups; the asterisk (*) represent a *p* < 0.0001 obtained with the Fisher exact test. RRHO plots comparing all gene expression data between LPS-Saline and Saline-Heroin (**D**); LPS-Saline and LPS-Heroin (**E**); or Saline-Heroin and LPS-Heroin (**F**). As indicated by white arrows, the upper right quadrant represents genes down-regulated in both groups while lower left quadrant represents genes up-regulated in both groups. **G** Venn diagram of DEGs regulated by LPS-Saline, Saline-Heroin, and/or LPS-Heroin in the NAc. Reds represents the number of genes co-regulated across all experimental groups. **H** Alluvial plot showing the flow of gene expression clusters through Saline-Heroin (S_H), LPS-Saline (LPS_S) and LPS-Heroin (LPS_H) conditions. Bar graphs represent Gene ontology (**I**) and KEGG pathway (**J**) analysis of co-regulated genes from LPS-Saline, Saline-Heroin, and LPS-Heroin conditions.

To better dissect the potential interplay between LPS-induced neuroinflammation and heroin exposure, we conducted an in-depth comparative transcriptomic analysis in the NAc across varying conditions of LPS and heroin exposure. Employing a combination of statistical methods and hypergeometric comparison, we identified significant overlaps in gene expression regulation across all experimental conditions. This integrative approach allowed for the identification of shared key genes, which in turn led to the identification of several pathways associated with addiction and neuroplasticity. In the comparative analysis between LPS-Saline and Saline-Heroin, 107 genes were commonly regulated in both datasets and significant overlap of the two datasets was observed (Fisher exact test *p* < 0.0001; Fig. 2A). Likewise, further analysis between LPS-Saline and LPS-Heroin identified a significant overlap in DEGs, with 183 genes commonly regulated (Fisher exact test *p* < 0.0001; Fig. 2B). Lastly, the comparison between Saline-Heroin and LPS-Heroin also yielded significant overlap in DEGs, with 203 genes commonly regulated (Fisher exact test, *p* < 0.0001; Fig. 2C). Complementing this findings, RRHO analysis demonstrated coordinated gene expression between these groups, revealing a striking similarity in regulation of gene activity between immune activation and heroin exposure, emphasizing the significance of shared regulatory mechanisms (Fig. 2D–F). Building on these findings, we next focused on identifying genes co-regulated across all the conditions under study, hypothesizing that these genes may play a pivotal role in inflammatory processes as well as in heroin’s responses. Our investigation revealed that 68 genes were consistently co-regulated in all three examined conditions, relative to saline-only treated rats (Fig. 2G). The alluvial plot complements this analysis by showing the flow of gene expression across conditions and revealing that the combined treatment of LPS-Heroin results in significant regulation of a large number of genes that were unregulated by LPS or Heroin alone (Fig. 2H and Table SE6). Analysis of the GO (BP) terms associated with the 68 commonly regulated genes highlighted a significant emphasis on neural processes and responses to environmental stimuli (Fig. 2I). Notably, the presence of terms related to glutamatergic synaptic transmission, such as “synaptic transmission, glutamatergic” and “negative regulation of synaptic transmission, glutamatergic”, along with “calcium ion transmembrane import into cytosol”, underscores the relevance of glutamatergic pathways and calcium-related processes in dual LPS-Heroin exposure (Fig. 2I). These pathways are crucial for neuroplasticity and learning, which are fundamental in the genesis of addiction and the modulation of inflammatory responses. Among the most represented genes in both GO (BP) and KEGG pathway analysis are the glutamate ionotropic receptor NMDA type subunits (*Grin2a* and *Grin2d)*, serotonin receptor 2A (*Htr2a*), and dopa decarboxylase (*Ddc*). Furthermore, the KEGG pathway analysis revealed a clear bias towards addiction-related pathways in the shared gene list, including “cocaine addiction,” “amphetamine addiction,” “alcoholism,” and “nicotine addiction” (Fig. 2J), thereby emphasizing the synergistic deleterious consequences of LPS-Heroin and providing rationale for studying the effects of inflammatory history on heroin-induced behavioral and molecular neuroadaptations in our preclinical rat model.

### Intermittent LPS pretreatment potentiates heroin-induced locomotor sensitization

**Fig. 3:**
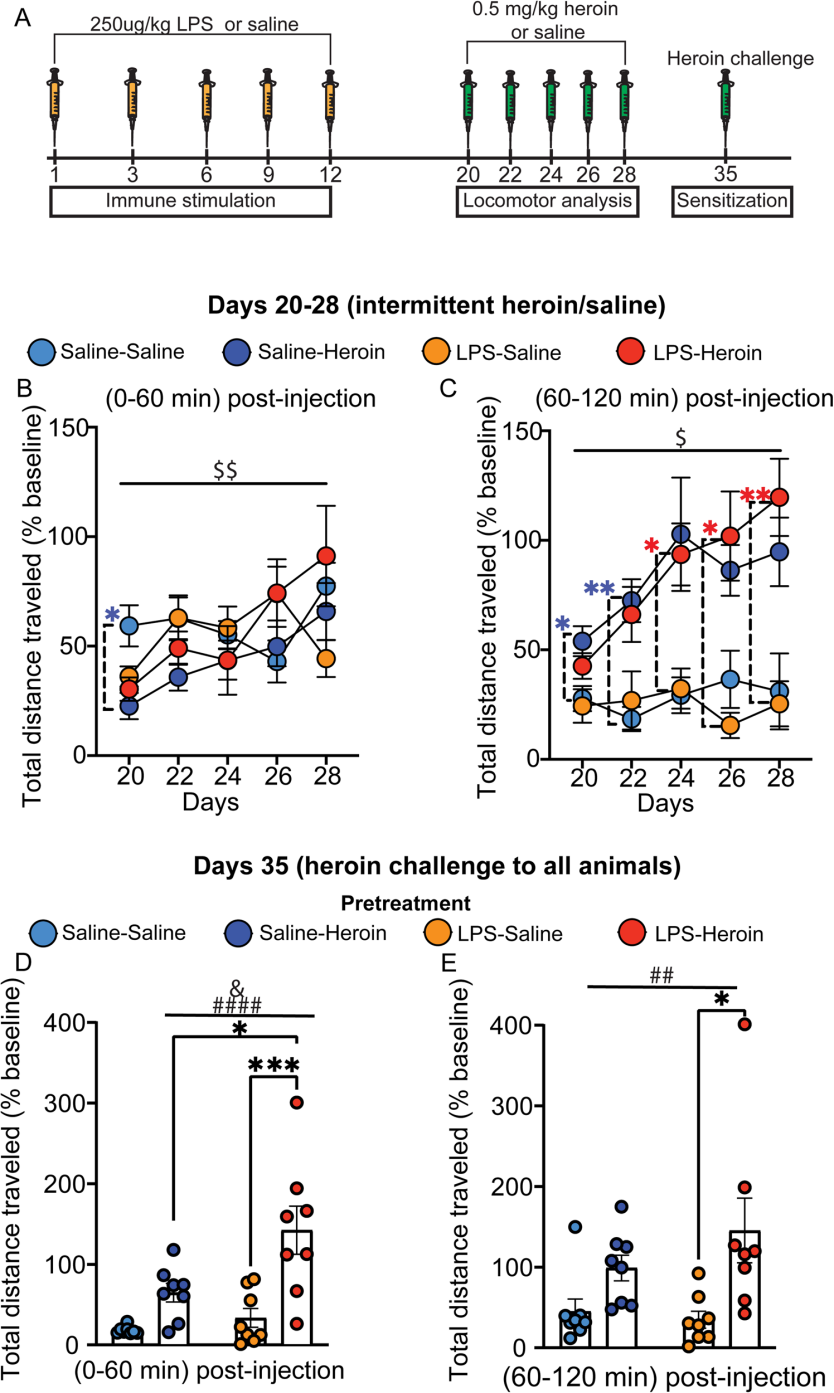
A history of subchronic LPS pretreatment heightens heroin-induced behavioral sensitization. **A** Schematic representation of heroin-induced locomotor sensitization experimental timeline. Rats received LPS or saline pretreatment every third day over 2 weeks. 8D later, animals received S.C. injections of heroin or saline every other day for 5 exposures. On D35, all rats were challenged with heroin. Locomotor activity was recorded for 1 h prior and 2 h following each heroin treatment and heroin challenge. Locomotor activity during the first (**B**) and second hours (**C**) following heroin injection. Time series line plot illustrating average of locomotor activity for each group (express in % of the baseline), measured over 5 distinct days. Each point represents the group average of locomotor activity for rats on a given day, connected by lines to depict trends over time. Post hoc test results are indicated with asterisk symbols (*) directly above individual data points. Blue asterisks denote significance between the Saline-Saline and Saline-Heroin groups, while red asterisks indicate significance between the LPS-Saline and LPS-Heroin groups. Error bars indicate mean ± S.E.M. Symbols above line indicate 2-way RM ANOVA: $ indicates interaction. Locomotor activity during the first (**D**) and second hours (**E**) following heroin challenge on day 35. Bar graphs represent total distance traveled (expressed in % of the baseline) and dots within the bars represent individual rats. Error bars indicate mean ± S.E.M. Symbols above line indicate 2-way ANOVA: # indicates main effect of heroin treatment; & indicates main effect of LPS pre-treatment; Post hoc test is indicated with asterisks symbols (*), directly above individual histograms. Irrespective of symbol types, one symbol indicates a *p* < 0.05; two symbols indicate a *p* < 0.01, three symbols indicate a *p* < 0.001, four symbols indicate a *p* < 0.0001. *n* = 8/group.

We next aimed to shed light on how pre-existing inflammatory states influence the neurobehavioral outcomes of heroin exposure, thereby providing a more comprehensive framework to explore the neurobiology of neuroinflammation in the context of heroin exposure. To accomplish this, we used a modified rat model of heroin-induced locomotor sensitization, where rats received subchronic LPS or saline injections over a 2-week period prior to heroin (Fig. 3A). This approach acknowledges the temporal and dose differences between our behavioral and transcriptomic analyses, emphasizing the importance of considering the behavioral evaluation as a method to study the functional consequences of prior immune activation in the context of heroin exposure. We recorded the total distance traveled for 1 h prior to heroin treatment on D20, 22, 24, 26, 28, and 35 to establish a baseline of locomotor movement, then for 2 h following heroin, as biphasic effects have been previously observed [25]. While variation in baseline locomotor behavior was observed on some days, no significant effects of LPS pretreatment or heroin treatment were observed during the baseline 1 h period prior to heroin (Fig. S4). Locomotor data during the period following heroin were normalized to baseline data for each day, represented as % baseline (Fig. 3), while raw total distance traveled can be found in the supplement (Fig. S5) and similar patterns were observed for both methods of data analysis. During the 0–60 min period post-heroin/saline injection, a two-way Repeated Measures (RM) ANOVA revealed a significant interaction between pretreatment (LPS or saline) and time (F (12, 112) = 2.381; *p* = 0.0090), as well as a significant main effect of time (F (3.139, 87.88) = 6.386; *p* = 0.0005; Fig. 3B). Tukey’s multiple comparisons test showed a significant change in locomotor activity between the Saline-Saline and Saline-Heroin groups (*p* = 0.0292) during this same 0–60 min period following heroin administration on D20, while no significant differences were found in the subsequent data points or between LPS-pretreated groups (Fig. 3B). During the second hour post-treatment, a two-way RM ANOVA detected a significant interaction between pretreatment and time (F (12, 112) = 1.983; *p* = 0.0321), a significant main effect of time (F (3.089, 86.50) = 5.703; *p* = 0.0012), and a significant main effect of pretreatment (F (3, 28) = 14.54; *p* < 0.0001; Fig. 3C). Tukey’s multiple comparisons test revealed a significant increase in locomotor activity in the Saline-Heroin group compared to the Saline-Saline group on D20 (*p* = 0.0482) and D22 (*p* = 0.0026), while no significant effects were found on subsequent days (Fig. 3C). In contrast, no significant difference in locomotor activity was observed on D20 and D22 when comparing the LPS-pretreated groups (LPS-Saline vs. LPS-Heroin), but significant differences emerged on D24 (*p* = 0.0158), D26 (*p* = 0.0145), and D28 (*p* = 0.0033; Fig. 3C). Following the five intermittent heroin/saline exposures, rats underwent 7D drug-free period in their homecage.

On D35, all rats were presented with a heroin challenge to evaluate the impact of LPS pretreatment on heroin-induced locomotor sensitization. During the first hour of the D35 challenge, the two-way ANOVA analysis revealed significant effects of both LPS pretreatment (F (1, 28) = 7.446, *P* = 0.0109) and heroin treatment (F (1, 28) = 20.93, *P* < 0.0001) on locomotor activity, with a trend for an interaction between these factors did not reach statistical significance (F (1, 28) = 3.337, *P* = 0.0784; Fig. 3D). The subsequent Tukey’s multiple comparisons test further delineated these effects, notably showing a significant difference between the LPS-Saline and LPS-Heroin groups (*P* = 0.0006), but no difference between Saline-Saline and Saline-Heroin groups (Fig. 3D). In the second hour on D35, two-way ANOVA showed a significant main effect of heroin treatment (F (1, 28) = 12.24, *P* = 0.0016), but no significant effects for interaction and pretreatment (Fig. 3E). Post hoc tests during the second hour of D35 indicated a significant difference between LPS-Saline and LPS-Heroin (*P* = 0.0124; Fig. 3E). In addition, we performed a further analysis for the entire duration of the experiment in the groups with the same experimental conditions from the beginning to the end of the study. The 2-way RM ANOVA revealed a significant interaction between pretreatment and time (F (5, 70) = 2.599; *p* = 0.0326), as well as a main effect of time (F (2.972, 41.60) = 10.53; *p* < 0.0001; Fig. S6B). Further analysis with Tukey’s multiple comparisons test showed a significant increase in locomotor activity in the LPS-Heroin group compared with the Saline-Heroin group following the drug-free period on challenge day (*p* = 0.0380) (Fig. S6B). On the other hand, we did not detect any statistical difference between these two groups during the 60–120 min period, but rather a main effect of time (F (2.694, 37.72) = 5.932; *p* = 0.0027), suggesting that at this time point, the effect is primarily driven by changes over time rather than by the experimental conditions (Fig. S6D). Regarding the control groups, LPS-Saline and Saline-Saline, no statistically significant differences were found (Fig. S6C, E). Together, these results demonstrate the significant influence of neuroinflammatory processes on heroin-induced behavioral outcomes.

### Overlap of DEGs in neuroinflammation and voluntary heroin exposure

**Fig. 4:**
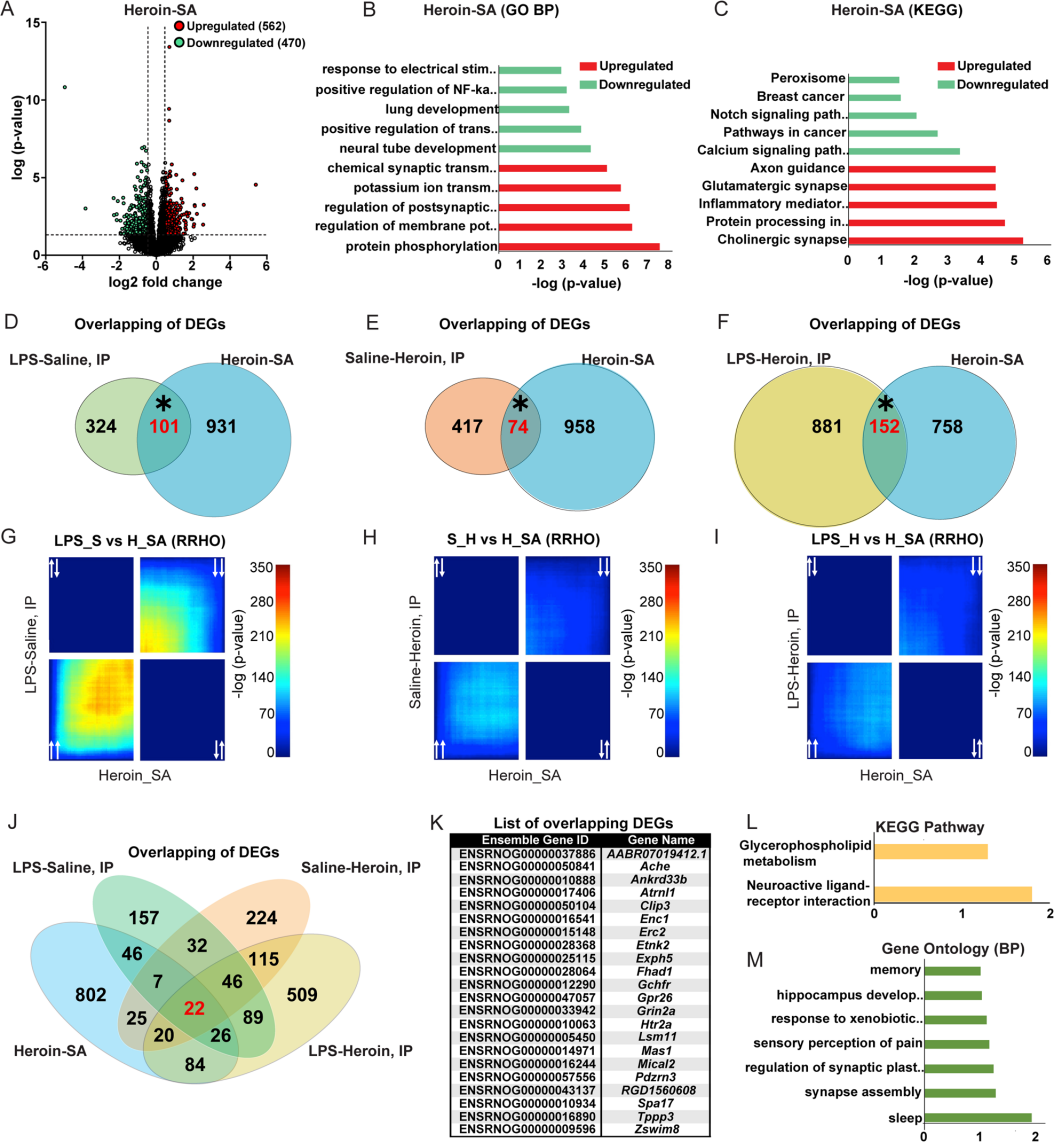
Identification of NAc transcripts commonly regulated by voluntary heroin intake and LPS pretreatment. **A** Volcano plot depicting differentially expressed genes (DEGs) in the NAc between Heroin self-administration (Heroin-SA) and saline-SA. Upregulated DEGs are represented in red, downregulated DEGs are represented in blue. Bar graphs representing GO BP (**B**) and KEGG (**C**) pathway analysis. Red bars represent significant pathways deriving from upregulated genes; blue bars represent significant pathways deriving from downregulated genes. Venn diagrams of total differential expressed genes (DEGs) regulated by LPS-Saline and/or Heroin-SA (**D**); Saline-Heroin and/or Heroin-SA (**E**); or LPS-Heroin and/or Heroin-SA (**F**) in the NAc. Red numbers represent genes co-regulated in both groups; the asterisk (*) represent a *p* < 0.0001 obtained with the Fisher exact test. RRHO plots comparing the NAc transcriptome between LPS-Saline and Heroin-SA (**G**); Saline-Heroin and Heroin-SA (**H**); or LPS-Heroin and Heroin-SA (**I**). White arrows represent gene expression directionality. Upper right quadrant represents genes down-regulated in both groups while lower left quadrant represents genes up-regulated in both groups. **J** Venn diagram of DEGs regulated by LPS-Saline, Saline-Heroin, LPS-Heroin and Heroin-SA in the NAc. Red numbers represent genes co-regulated in both groups. **K** Table of the 22 co-regulated genes from (**J**). Bar graphs representing KEGG (**L**) and GO BP (**M**) pathway analysis of the 22 co-regulated genes from (**J**).

In our effort to reveal NAc transcriptional signatures associated with heroin exposure that are shared by LPS-induced inflammation, we extended the transcriptomic analysis to a rat model of heroin self-administration (Heroin-SA). By including this new dataset, we conducted further comparisons with the other experimental groups (Heroin-SA vs. LPS-Saline, Saline-Heroin, and LPS-Heroin). This approach allowed us to directly compare the transcriptional changes across different contexts of heroin exposure, gaining insights into the specific transcriptional changes that are consistent across voluntary and experimenter-administered models of heroin exposure, as well as across neuroinflammation. Rats underwent Heroin-SA for 10D and displayed a significant preference for the drug-paired active lever compared to an inactive lever (Fig. S7). Following Heroin-SA, 1032 DEGs were observed in the NAc compared to saline SA rats, with 562 upregulated and 470 downregulated (Fig. 4A and Table SE7). Further analysis of NAc Heroin-SA DEGs through Gene Ontology (GO) analysis revealed terms such as protein phosphorylation, regulation of membrane potential, regulation of postsynaptic membrane potential, and potassium ion transmembrane potential (Fig. 4B). The KEGG pathway analysis provided insights into the specific pathways impacted by heroin-SA, including cholinergic synapse, glutamatergic synapse, calcium signaling pathway and Inflammatory mediator regulation of TRP channels (Fig. 4C). When comparing Heroin-SA across all experimental groups, with a special emphasis on LPS-Heroin and LPS-Saline due to their similarities, we observed a commonality in the calcium signaling pathways. Additionally, there was a convergence on terms associated with the regulation of membrane potential within the LPS-Heroin group and a shared emphasis on neurotransmission pathways with the LPS-Saline group (Figs. 2 and 4). Conversely, there was minimal overlap with the Saline-Heroin group, with axon guidance being the only common pathway. We observed a significant overlap of DEGs between Heroin-SA and LPS-Saline, Saline-Heroin and LPS-Heroin groups (Fisher’s exact test, *p* < 0.0001; Fig. 4D–F). 101 genes were co-regulated by Heroin-SA and LPS-Saline groups, while only 74 were in common between Heroin-SA and Saline-Heroin (Fig. 4D, E). The highest number of commonly regulated genes were between Heroin-SA and LPS-Heroin groups, with 152 genes (Fig. 4F). In contrast, RRHO analysis of NAc transcriptomes revealed moderate or little overlapping between Heroin-SA and other experimental groups, with the most overlap observed between Heroin-SA and LPS-Saline, further highlighting the similarity at the transcriptional level associated with heroin intake and immune activation, and indicating that the NAc transcriptome organization changes between Heroin-SA and experimenter-administered heroin exposure (4G-I). Finally, we overlapped all DEGs and found that 22 genes that were common in all datasets (Fig. 4J). Specifically, the co-regulated DEGs were: *AABR07019412.1*, *Ache*, *Ankrd33b, Atrnl1, Clip3, Enc1, Erc2, Etnk2, Exph5, Fhad1, Gchfr, Gpr26, Grin2a, Htr2a, Lsm11, Mas1, Mical2, Pdzrn3, RGD1560608, Spa17, Tppp3*,and *Zswim8* (Fig. 4K). We then used this list for pathway analysis and observed an enrichment of terms related to synaptic plasticity and memory formation in the GO (BP) analysis for the overlapping gene list (Fig. 4M), as well as the recurrent “Neuroactive ligand-receptor interaction” pathway and the Glycerophospholipid metabolism pathway in the KEGG pathway analysis (Fig. 4L).

### Overlap of DEGs between LPS-Heroin and human OUD

**Fig. 5:**
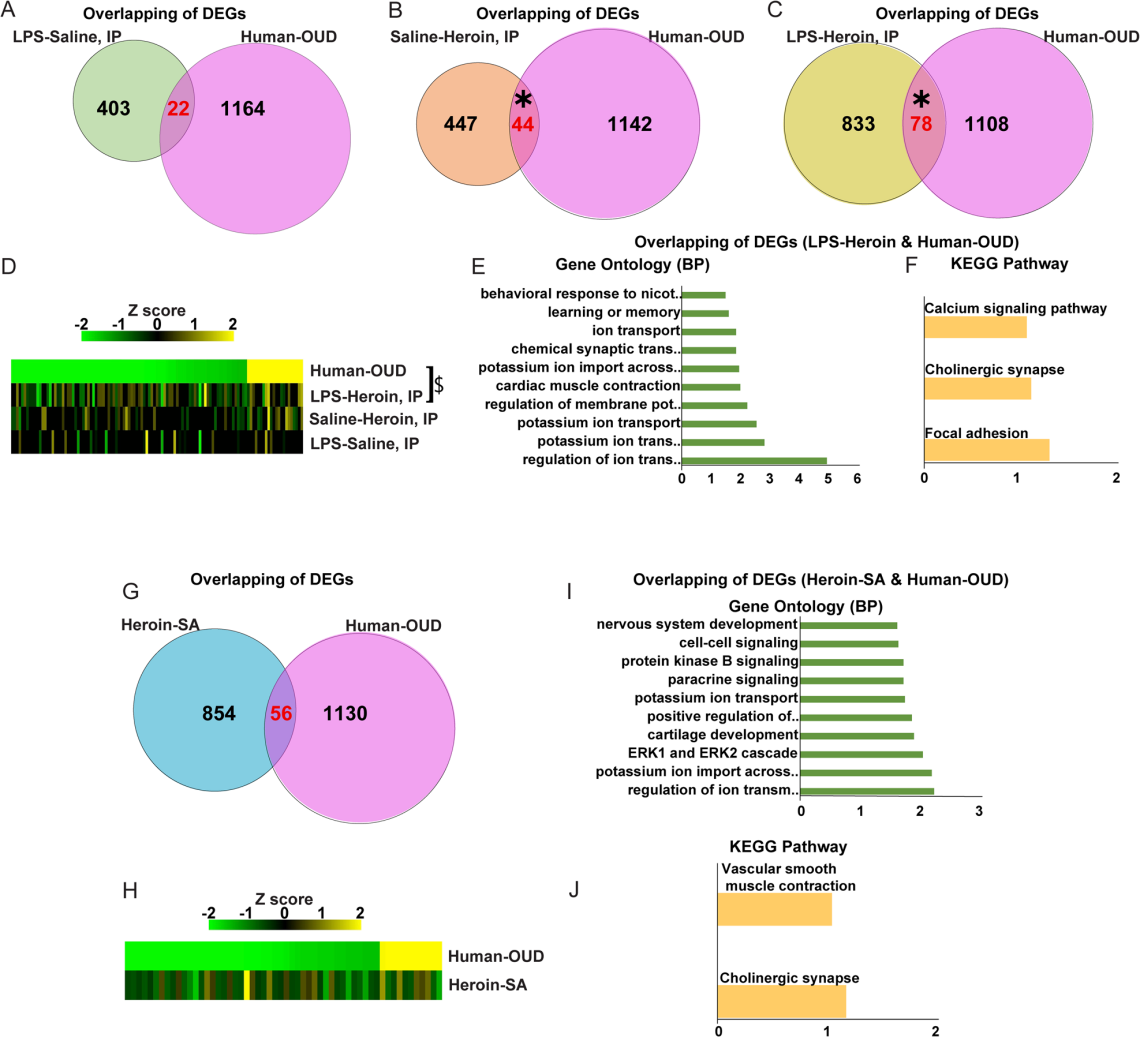
Rat NAc LPS-heroin transcriptome significantly overlaps with Human OUD NAc transcriptome dataset. Venn diagrams of total differential expressed genes (DEGs) regulated by LPS-Saline (**A**), Saline-Heroin (**B**), LPS-Heroin (**C**) and/or Human OUD from Seney et al. (2021) in the NAc. Red numbers represent the genes co-regulated in both groups; the asterisk (*) represent a *p* < 0.0001 obtained with the Fisher exact test. **D** Union heatmaps illustrating the Pearson gene expression correlations in Human OUD subjects (upper row, with down-regulation shown in green and up-regulation in yellow) and the three experimental rat conditions (LPS-Saline, Saline-Heroin, and LPS-Heroin); (z-score absolute value > 0.2, P-value < 0.05); $ symbol represent Pearson correlation coefficient (*p* = 0.0496). Bar graphs represent Gene Ontology (BP) (**E**) and (KEGG) pathway analysis (**F**) of the 78 co-regulated DEGs between LPS-Heroin, and Human-OUD. **G** Venn diagram of total DEGs regulated by Heroin-SA and/or Human-OUD in the NAc. **H** Union heatmaps illustrating the gene expression correlations in Human-OUD (upper row, with down-regulation shown in green and up-regulation in yellow) and Heroin-SA. Bar graphs represent GO (BP) (**I**) and KEGG pathway analysis (**J**) of the 56 co-regulated DEGs between LPS-Heroin and Human-OUD condition.

Following the analysis of the heroin-SA data, we proceeded to integrate the analysis of our datasets with a published human OUD transcriptomic dataset obtained from the NAc of postmortem tissue samples [4]. 22 DEGs were common between LPS-Saline and Human OUD datasets but no significant overlap was observed with a Fisher’s exact test (Fig. 5A and Tables SE8, 9). 44 DEGS were commonly expressed between Human OUD and the Saline-Heroin group, and 78 with the LPS-Heroin group, with significant overlap between Human OUD and both of these rat model groups (Fisher’s exact test: *p* < 0.0001; Fig. 5B, C and Tables SE8, 9). Pearson correlation coefficient assessing the linear correlation across datasets revealed a modest, yet significant correlation between LPS-Heroin and Human OUD (*p* = 0.04956) and a strong trend between Saline-Heroin and Human OUD (*p* = 0.0607), while the Pearson correlation analysis between LPS-Saline and Human OUD was not significant (*p* = 0.3897; Fig. 5D and Tables SE8, 9). RRHO plots were generated to compare between Human OUD data and our rat LPS and/or Heroin datasets, and can be found in Supplemental Fig. S8. The analysis revealed no concordance between the human OUD and the rat datasets, highlighting distinct mechanisms of overall gene expression orchestration across species, as has been reported in other similar studies [34].

Pathway analysis of the 78 commonly regulated DEGs between Human OUD and LPS-Heroin datasets revealed GO (BP) terms related to ion transport, in particular potassium, while the KEGG pathways “Focal adhesion” “Cholinergic synapse” and “Calcium signaling pathway” were found to be significant (Fig. 5E, F). Next, we also compared our heroin-SA data with the Human OUD dataset, and we found that 56 DEGs were commonly regulated between these two conditions (Fig. 5G, H and Tables SE8, 10). Moreover, GO (BP) and KEGG pathway analysis with the 56 commonly regulated DEGs lead to similar results obtained with the intersection between LPS-Heroin and Human OUD, including terms related to ion and potassium transport and the “Cholinergic synapse” common between the two (Fig. 5I, J). The identification of potassium channel genes KCNJ16, KCNJ4, KCNK12, KCNH2, KCNH3, KCNH8, KCNJ12, and KCNQ2 as enriched in both comparisons underscores the critical role of these channels’ pathways in OUD [35].

## Discussion

The intricate relationship between opioids and the brain’s immune system response presents a compelling intersection of neuroscience and immunology, reflecting a burgeoning field of research aimed at elucidating the underlying mechanisms of OUD [2]. Our study advances this exploration by documenting that neuroinflammatory responses elicited by subchronic LPS administration result in gene expression alterations mirroring those triggered by heroin exposure, suggesting an intimate relationship between these conditions. We found a robust and consistent coordination of gene expression across LPS-Saline, Saline-Heroin and LPS-Heroin conditions as indicated by RRHO plots. This pattern of transcriptional coordination was further validated at the level of differentially expressed genes using Fisher’s exact test, demonstrating a substantial overlap across our datasets. Furthermore, pathway analysis arising from the overlap of DEGs common to all datasets led to the discovery of four out of six pathways with direct reference to addiction-related terms. Additionally, the “neuroactive ligand-receptor interaction” pathway was highlighted as central in a comprehensive meta-analysis exploring common molecular pathways identified in different types of drug addiction, and in a genome-wide gene-set analysis aimed to identify pathways associated with alcohol dependence [36, 37]. This further indicates shared molecular profiles between between neuroinflammation and heroin exposure.

These intriguing findings indicate that heroin exposure may trigger pathways that overlap with those activated by immune stimulation, or alternatively, it may directly affect the immune system, which is ultimately responsible for producing these changes. It has been suggested that opioids can provoke a neuroinflammatory response through the direct stimulation of TLR4, a membrane receptor for bacterial LPS [38–40]. Regardless of the specific mechanism, there exists a multitude of ways through which opioids might initiate inflammatory phenomena involving this receptor [38], which is consistent with our transcriptomic data showing a significant overlap between animals exposed to heroin and LPS. Supporting this theory, there is growing evidence that opioids can directly interact with the immune system through several mechanisms [39, 41, 42]. While the overall effect of opioids on the immune system appear to be anti-inflammatory [38], a substantial body of experimental articles in rodents demonstrates upregulation and/or enhanced release of cytokines in different brain areas that regulate reward including the NAc [12, 43, 44]. This contradiction underscores the complexity of opioids’ impact on inflammatory responses which likely varies based on factors such as the method of drug administration, frequency and duration of exposure, differences across species, and the occurrence of specific localized interactions in various parts of the brain [2]. Interestingly, a recent transcriptomic analysis of patients with OUD has led to the conclusion that chronic opioid use may trigger an inflammatory state in the brain. This model suggests that opioid-induced neuroinflammation could be the catalyst for the synaptic changes that ultimately form the foundation of opioid dependence [4]. Remarkably, when comparing our datasets with human OUD dataset from Seney et al. [4], we found a robust and significant overlap with the LPS-Heroin group. However, only the Fisher exact test yielded significant results for the Saline-Heroin group, whereas the LPS-Saline group showed no significant findings. This discrepancy suggests the necessity of combined immune responses from LPS and heroin to accurately simulate the chronic inflammation observed in OUD patients. Accordingly, behaviors associated with opioid dependence, such as needle sharing, may exacerbate systemic inflammation in OUD individuals [45]. It is important to note the exposure duration to LPS and heroin was relatively brief. Consequently, this short exposure may not fully replicate the chronic neuroinflammation to which patients with OUD may be subjected. Thus, a group in which the treatment with LPS is followed by heroin SA may serve as a better model for recapitulating the molecular changes observed in OUD human patients.

In our study, gene expression was measured nearly two weeks after the last LPS injection, a timing chosen to assess the long-term rather than acute transcriptional effects of LPS. While initial responses to LPS are characterized by a surge in immune-related gene activity [32], these effects typically subside, leading to regulation of a broader range of gene categories. Our findings indicate that the enduring effects of LPS exposure extend beyond traditional immune pathways to influence a diverse set of genes, including those involved in cellular metabolism, synaptic function, and neurotransmitter receptor regulation. This suggests a complex interplay between early inflammatory responses and subsequent, sustained modifications to brain circuitry, potentially influencing sensitivity to subsequent heroin exposure. In this context, our behavioral results, highlighting a marked increase in heroin-induced locomotor sensitization exclusively in the group pre-treated with LPS, further support this hypothesis. Thus, it is conceivable that an initial immune challenge may amplify subsequent responses to opioids, suggesting a primed neural state conducive to enhanced sensitivity to the drug. This primed state is likely due to the fact that many transcriptional variations resulting from neuroinflammation are common in animals exposed to heroin. Our behavioral data are supported by a recent study showing that a similar LPS regimen increased morphine-induced conditioned place preference [46]. While the conditioned place preference paradigm highlights how LPS amplifies the rewarding effects of morphine, our study on locomotor sensitization offers a different perspective on the temporal progression and dynamics of this phenomenon. For example, the attenuated dopaminergic response to heroin observed in the early phase post-LPS pretreatment, as suggested by decreased locomotor activity, is particularly interesting. It suggests a scenario where neuroinflammation initially imposes a negative regulation on dopaminergic transmission. This, however, may set the stage for a subsequent state of enhanced dopamine sensitivity, potentially heightening the dopaminergic system’s responsiveness to opioids, such us during the heroin challenge after the drug-free period. It is thus possible that a hypodopaminergic state, produced by an inflammatory response, may serve as a vulnerability factor for facilitating the propensity for drug addiction [47, 48]. This hypothesis agrees with existing human literature showing that innate immune activation and subsequent inflammatory cytokine release predominantly impact reward circuitry and basal ganglia dopamine, leading to diminished motivation and motor activity [49–52]. Furthermore, preclinical studies in non-human primates and rodents corroborate that inflammation-induced alterations in striatal dopamine are associated with the observed changes in reward circuitry and motivation [53–55].

Another interesting finding of our paper is the overlap of our datasets from LPS and experimental heroin exposure with animals that self-administered heroin voluntarily. We found a significant overlap at the DEG level across all groups that indicates a shared molecular basis underlying both involuntary exposure and voluntary consumption of heroin, integrated within the context of inflammation. However, this level of overlapping was not maintained when we look at the overall coordination of transcriptional responses (RRHO plot). Despite this, a certain level of coordination of transcriptional response between the LPS-saline and Heroin-SA groups was present. Together, these findings underscore the profound impact of heroin exposure on neuroinflammation, bridging forced and voluntary drug experiences through similar transcriptomic alterations.

Despite identifying 56 DEGs commonly regulated between our heroin-SA dataset and the Human-OUD dataset from Seney et al. [4], we observed no significant overlap with the Fisher exact test. This discrepancy may be attributed to several factors, notably the limited duration of Heroin-SA (10 days) in our experimental design, which does not include any withdrawal phases. Bridging this gap, Browne et al. [34] demonstrated a significant overlap in NAc data from mice subjected to a short-term withdrawal period (24 h) following a more extended period of 15 days of opioid SA, when compared to the Seney datasets. This finding suggests that a longer duration of Heroin-SA, incorporating the withdrawal period, may be important for better replicating the complex gene expression patterns characteristic of human OUD. Indeed, considering that samples from the NAc of OUD patients were collected post-mortem following death due to overdose—and thus not from individuals during withdrawal periods—these samples likely represent individuals who had experienced multiple withdrawal periods throughout their history of drug dependence. This suggests the necessity of incorporating periods of withdrawal in experimental models to more accurately reflect the human experience and increase the reliability of the transcriptional profiles observed in animal models of chronic opioid use. This interpretation aligns with research indicating that opioid withdrawal may facilitate the translocation of Gram-negative bacteria and lipopolysaccharides from the intestinal lumen, potentially contributing to the sustained inflammatory conditions observed in human OUD [38]. Regardless of these nuances, our findings shed light on the transcriptional links between neuroinflammation and heroin exposure, while also highlighting the behavioral consequences of these interactions. By elucidating the connection at the transcriptional level between neuroinflammation and heroin exposure, our research offers critical insights that could guide future studies in reducing the impact of inflammatory pathways on OUD.

## Supplementary information

Supplemental Material

Supplemental Tables

## Data Availability

Data are available from the corresponding author on reasonable request.
