# Supplementary material for: Neuroinflammatory history results in overlapping transcriptional signatures with heroin exposure in the nucleus accumbens and alters responsiveness to heroin in male rats
Source: Transl Psychiatry. 2024 Dec 19;14:500. doi: 10.1038/s41398-024-03203-4 (PMC11659471; doi:10.1038/s41398-024-03203-4)
Supplement: Supplementary file 1 — Supplemental Material [file 41398_2024_3203_MOESM1_ESM.pdf]

Supplemental information for “Neuroinflammatory history results in overlapping transcriptional signatures with heroin exposure in the nucleus accumbens and alters responsiveness to heroin in male rats” by Floris et al.

Supplemental methods

RNA sequencing:

RNA sequencing (RNA-seq) analyses were performed by Novogene on the Illumina NovaSeq 6000 platform. Messenger RNA was purified from total RNA using poly-T oligo-attached magnetic beads. After fragmentation, first strand cDNA was synthesized using random hexamer primers, followed by second strand cDNA synthesis using dUTP. The directional library was prepared by performing end repair, A-tailing, adapter ligation, size selection, USER enzyme digestion, amplification, and purification. The library was checked with Qubit and real-time PCR for quantification and a Bioanalyzer was used for size distribution detection. Clustering of the index-coded samples was performed according to the manufacturer’s instructions. After cluster generation, the library preparations were pooled and sequenced to obtain paired-end reads. Raw data in FASTQ format were first processed through fastp. Clean reads were obtained by removing reads containing adapters, poly-N sequences and reads with low quality. Reference genome and gene model annotation files were downloaded from the genome website browser (NCBI/UCSC/Ensembl) directly. Paired-end clean reads were mapped to the reference genome using HISAT2 software (Langmead, Wilks et al. 2019). HISAT2 uses a large set of small GFM indexes that collectively cover the whole genome to enable rapid and accurate alignment of sequencing reads. Featurecounts (Liao, Smyth et al. 2014) was used to count the read numbers mapped for each gene. RPKM, Reads Per Kilobase of exon model per Million mapped reads, of each gene was calculated based on the length of the gene and reads count mapped to the

gene. Differential expression analysis between two conditions/groups was performed using the DESeq2 R package (Love, Huber et al. 2014). The resulting P values were adjusted using the Benjamini and Hochberg's approach for controlling the False Discovery Rate (FDR). Genes with an adjusted P value < 0.05 found by DESeq2 were assigned as differentially expressed.

Bioinformatic analysis:

Gene Ontology (GO) and KEGG enrichment analysis of differentially expressed genes were implemented with the DAVID Bioinformatic software by NIH (Huang da, Sherman et al. 2009). Terms with corrected $p<0.05$ were considered significantly enriched by differential expressed genes. The RRHO2 package optimized by Dr. Li Shen's lab (github.com/shenlab-sinai/RRHO2) was used to generate the stratified RRHO plots. In order to perform the pattern analysis, genes across experimental conditions were categorized as not significant (ns), and up- or downregulated using p-value ≤ 0.05 and Log2FoldChange ≥ 0.378 or p-value value ≤ 0.05 and Log2FoldChange ≤ -0.378 respectively. Alluvial plots were generated using the ggalluvial package in R (version 0.12.5). The significance of the overlap between any two lists of significantly regulated genes (p-value ≤ 0.05 and Log2FoldChange ≥ 0.378 or Log2FoldChange ≤ -0.378) were calculated via Fisher's exact test using GeneOverlap package in R Bioconductor, version 1.36.0.

Comparison with human OUD RNA-seq dataset

Human RNA-seq data from the Nucleus Accumbens were accessed from Seney et al. (Seney, Kim et al. 2021). Z-scores were calculated to compare our rat-generated RNA-seq datasets with the human NAc OUD gene expression data. Significantly up- and downregulated genes (p-value ≤ 0.05 and Log2FoldChange ≥ 0.378 or Log2FoldChange ≤ -0.378) across the datasets were merged. Two union heatmaps were generated: 1) human NAc, rat LPS-Heroin vs Saline-Saline,

rat Saline-Heroin vs Saline-Saline, rat LPS-Saline vs Saline-Saline and 2) human NAc, rat Heroin-SA vs Saline-SA to identify patterns of convergence between conditions. Pearson correlations were performed to evaluate the significance of the correlations between the transcriptional signatures associated with each experimental group. BioMart data mining tool from Ensembl was used identify Ensembl IDs of rat orthologs for the human data in order to compare the human transcriptomic profile with the rat transcriptomic profile using RRHO2 analyses.

Supplemental Figure 1: Subchronic LPS treatment induces a mild and transitory alteration of rat's body weight.

Body weight of rats during a subchronic IP LPS injection paradigm. Arrows indicate LPS injections. N=16/group. (A) Depicted are weights of animals used for the behavioral assessment of heroin-induced locomotor sensitization. (B) Weights of animals used for RNA-seq analysis.

A.

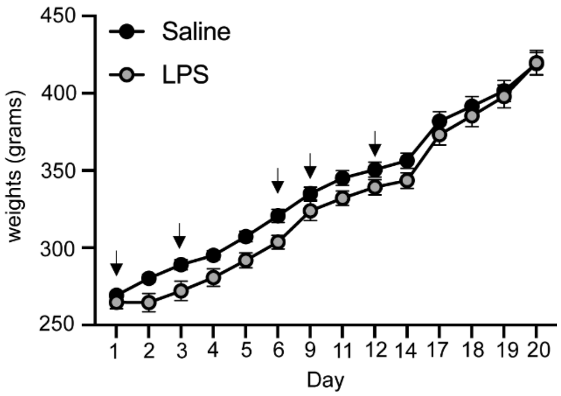

B.

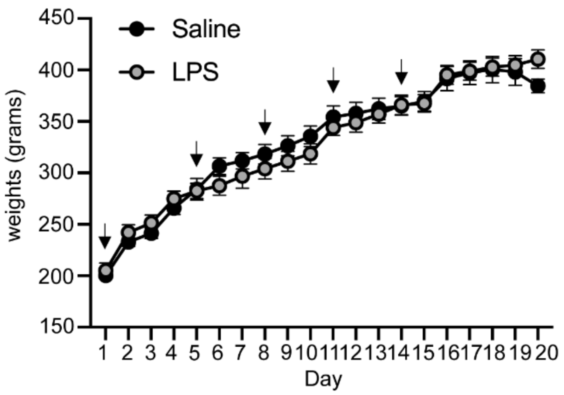

Supplemental Figure 2: Locomotor data for animals that underwent RNA-seq

The total distanced traveled during the first 30 min following heroin treatment for animals that underwent RNA-seq in Fig. 1. Black dots represent individual values within Saline-Saline (S-S) group. Green dots represent individual values within LPS-Saline (L-S) group. Blue dots represent individual values within Saline-Heroin (S-H) group. Red dots represent individual values within LPS-Heroin (L-H) group. One way ANOVA reveal a main effect of treatment $F(3, 26) = 4.425$; $p=0.0122$, which is indicated with asterisk symbol (*). Error bars indicate mean +/- S.E.M.

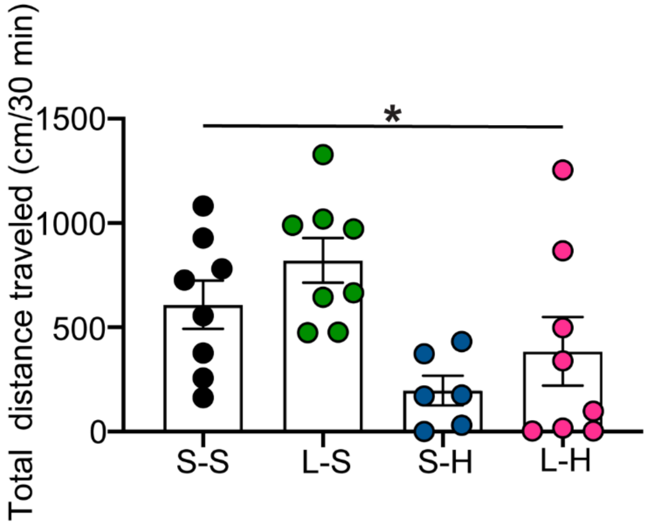

Supplemental Figure 3: RNA-seq transcriptome analysis following LPS and heroin identifies heroin-regulated genes in the NAc potentiated by LPS. (A) Experimental design of RNA-seq study in the NAc tissue with experimental groups. (B) Schematic representation of experimental timeline. Rats received LPS or saline every third day. Four days later, rats received injection of heroin or saline for five consecutive days. (C-H) Volcano plots (C,F) depicting differentially expressed genes (DEGs) in the NAc and bar graphs depicting Gene Ontology (D, G) or KEGG pathways analysis (E, H) of significantly altered pathways between LPS-Heroin and: Saline-Heroin (C, D, E) or LPS-Saline treated rats (F, G, H). Upregulated DEGs and pathways are represented in red, downregulated DEGs and pathways are represented in green.

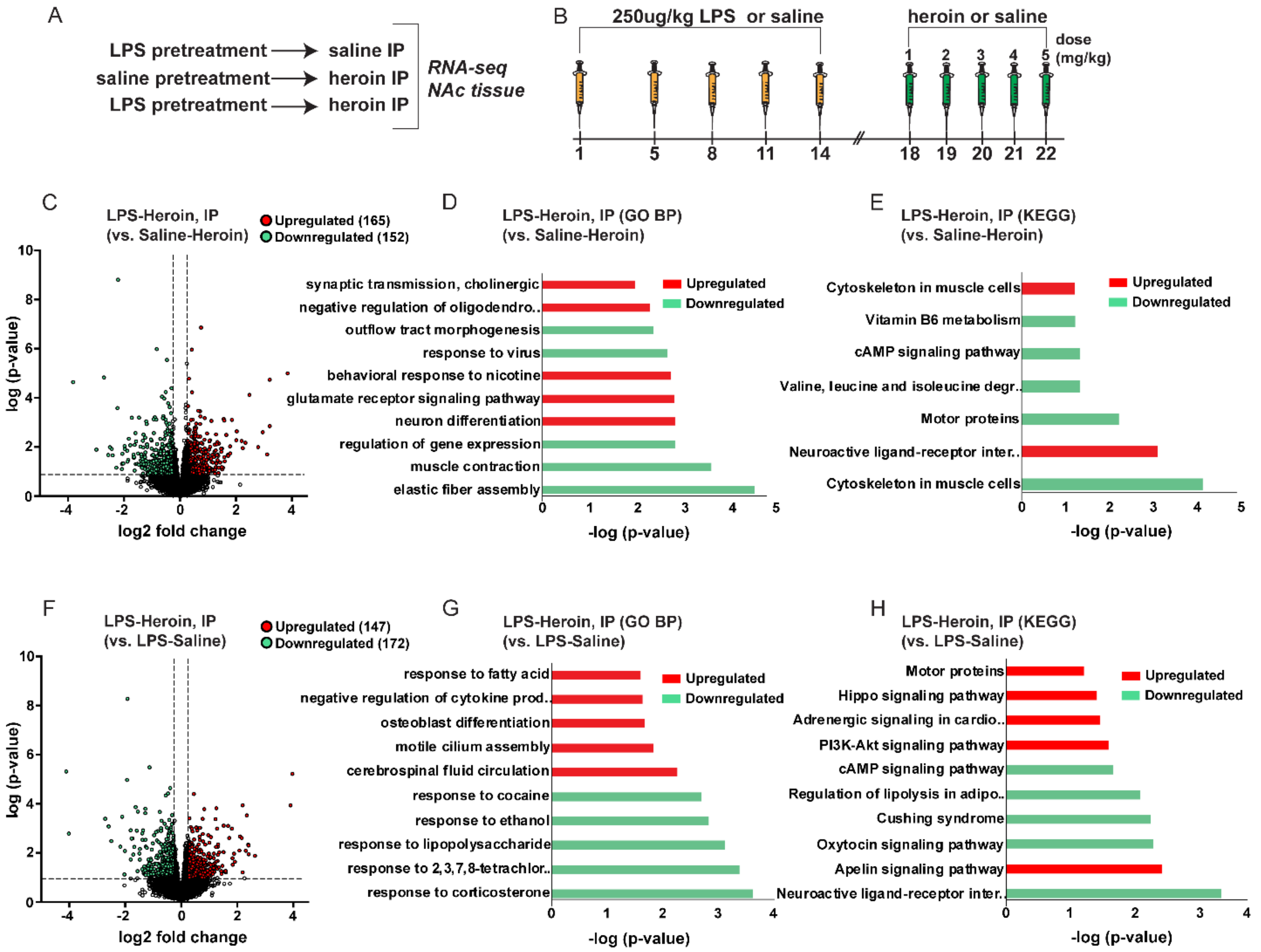
A
LPS pretreatment → saline IP
saline pretreatment → heroin IP
LPS pretreatment → heroin IP
RNA-seq NAc tissue
B
250ug/kg LPS or saline
heroin or saline
dose (mg/kg)
1 5 8 11 14 18 19 20 21 22
C
LPS-Heroin, IP (vs. Saline-Heroin)
Upregulated (165)
Downregulated (152)
log (p-value)
log2 fold change
D
LPS-Heroin, IP (GO BP) (vs. Saline-Heroin)
synaptic transmission, cholinergic
negative regulation of oligodendro..
outflow tract morphogenesis
response to virus
behavioral response to nicotine
glutamate receptor signaling pathway
neuron differentiation
regulation of gene expression
muscle contraction
elastic fiber assembly
Upregulated
Downregulated
-log (p-value)
E
LPS-Heroin, IP (KEGG) (vs. Saline-Heroin)
Cytoskeleton in muscle cells
Vitamin B6 metabolism
cAMP signaling pathway
Valine, leucine and isoleucine degr..
Motor proteins
Neuroactive ligand-receptor inter..
Cytoskeleton in muscle cells
Upregulated
Downregulated
-log (p-value)
F
LPS-Heroin, IP (vs. LPS-Saline)
Upregulated (147)
Downregulated (172)
log (p-value)
log2 fold change
G
LPS-Heroin, IP (GO BP) (vs. LPS-Saline)
response to fatty acid
negative regulation of cytokine prod..
osteoblast differentiation
motile cilium assembly
cerebrospinal fluid circulation
response to cocaine
response to ethanol
response to lipopolysaccharide
response to 2,3,7,8-tetrachlor..
response to corticosterone
Upregulated
Downregulated
-log (p-value)
H
LPS-Heroin, IP (KEGG) (vs. LPS-Saline)
Motor proteins
Hippo signaling pathway
Adrenergic signaling in cardio..
PI3K-Akt signaling pathway
cAMP signaling pathway
Regulation of lipolysis in adipo..
Cushing syndrome
Oxytocin signaling pathway
Apelin signaling pathway
Neuroactive ligand-receptor inter..
Upregulated
Downregulated
-log (p-value)

Supplemental Figure 4: First hour baselines of heroin-induced locomotor sensitization assay

The total distanced traveled during the first hour (baseline), prior to heroin treatment for each day that locomotor behavior was assessed during the heroin-induced locomotor sensitization assay, as described in Figure 3. Black dots represent individual values within Saline-Saline (S_S) group. Green dots represent individual values within LPS-Saline (L_S) group. Blue dots represent individual values within Saline-Heroin (S_H) group. Red dots represent individual values within LPS-Heroin (L_H) group. No significant differences in baseline locomotor behavior were observed between treatment groups for any day. Error bars indicate mean +/- S.E.M.

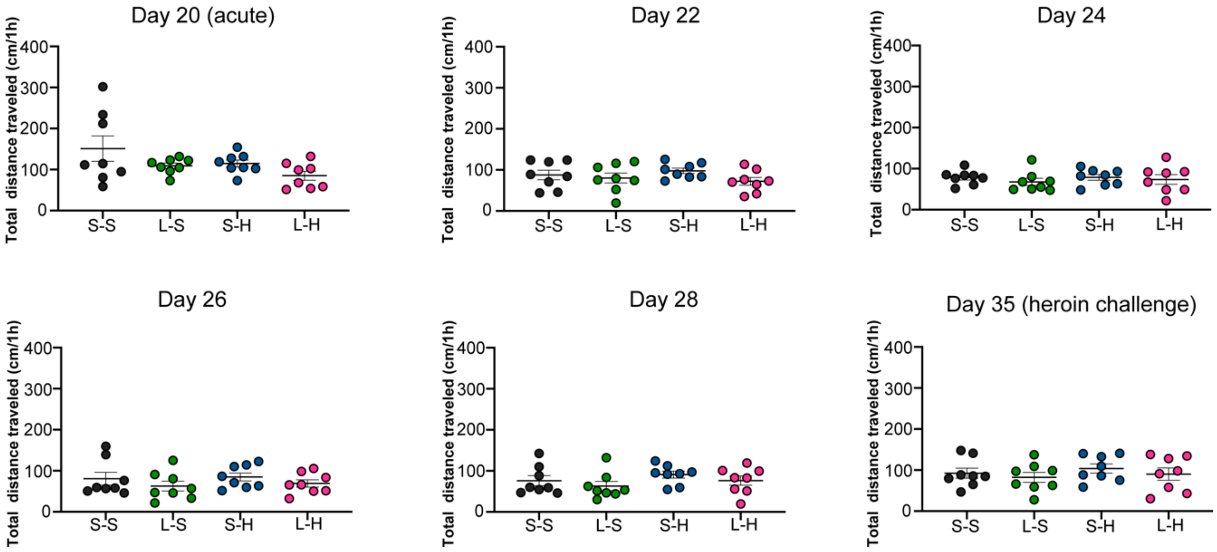

Supplemental Figure 5: Subchronic LPS pretreatment exacerbates heroin-induced locomotor sensitization.

Locomotor activity during the first (left panel) and second hour (h; right panel) following heroin injection during the heroin-induced locomotor sensitization assay, as described in Figure 3. Bar graphs represent total distance traveled (cm/1h). Error bars indicate mean +/- S.E.M. Symbols above line indicate 2-way ANOVA: # indicates main effect of heroin treatment; & indicates main effect of LPS pre-treatment; Post hoc test is indicated with asterisk symbols (*), directly above individual histograms. Irrespective of symbol types, one symbol indicates p-value <0.05; two symbols indicate p-value <0.01, three symbols indicate a p-value <0.001, four symbols indicate a p-value <0.0001. Orange dots represent individual animals assigned to the saline group during the “locomotor analysis period” while blue dots represent individual animals assigned to the heroin group during the “locomotor analysis period”. *n*=8/group.

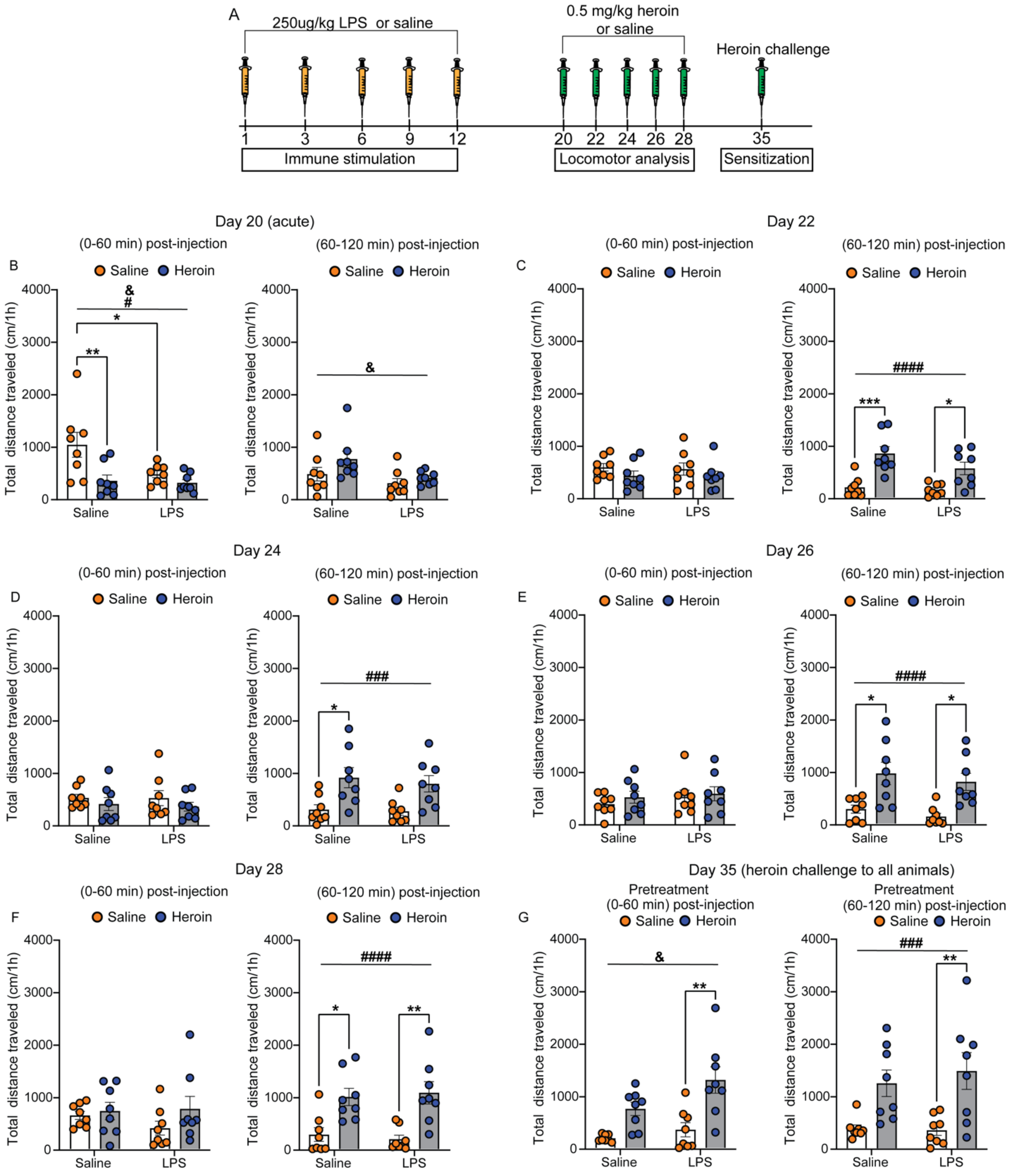

A
250ug/kg LPS or saline
0.5 mg/kg heroin or saline
Heroin challenge
1 3 6 9 12 20 22 24 26 28 35
Immune stimulation
Locomotor analysis
Sensitization
Day 20 (acute)
Day 22
Day 24
Day 26
Day 28
Day 35 (heroin challenge to all animals)
(0-60 min) post-injection
(60-120 min) post-injection
Pretreatment
B
C
D
E
F
G
Saline
Heroin
Total distance traveled (cm/1h)
Saline
LPS

Supplemental Figure 6: Increased heroin-induced locomotor sensitization in rats pretreated with LPS during the first 60 minutes post-heroin challenge

(A) Schematic representation of heroin-induced locomotor sensitization experimental timeline. Rats received LPS or saline pretreatment every third day over 2 weeks. 8D later, animals received S.C. injections of heroin or saline every other day for 5 exposures. On D35, all rats were challenged with heroin. Locomotor activity was recorded for 1h prior and 2h following each heroin treatment and heroin challenge. (B-E) Locomotor analysis of the total distance traveled (% baseline) in the first hour (B, C) or second hour (D, E) post injection on days 20-35. Rats in B & D received heroin each day on days 20-35. Rats in C & E received saline on days 20-28 then a heroin challenge on day 35. for rats that received heroin. Error bars indicate mean +/- S.E.M. Symbols above line indicate 2-way RM ANOVA: $ indicates interaction; ^ indicates main effect of time. Post hoc test results are indicated with asterisk symbols (*) directly above individual data points. *n*=8/group.

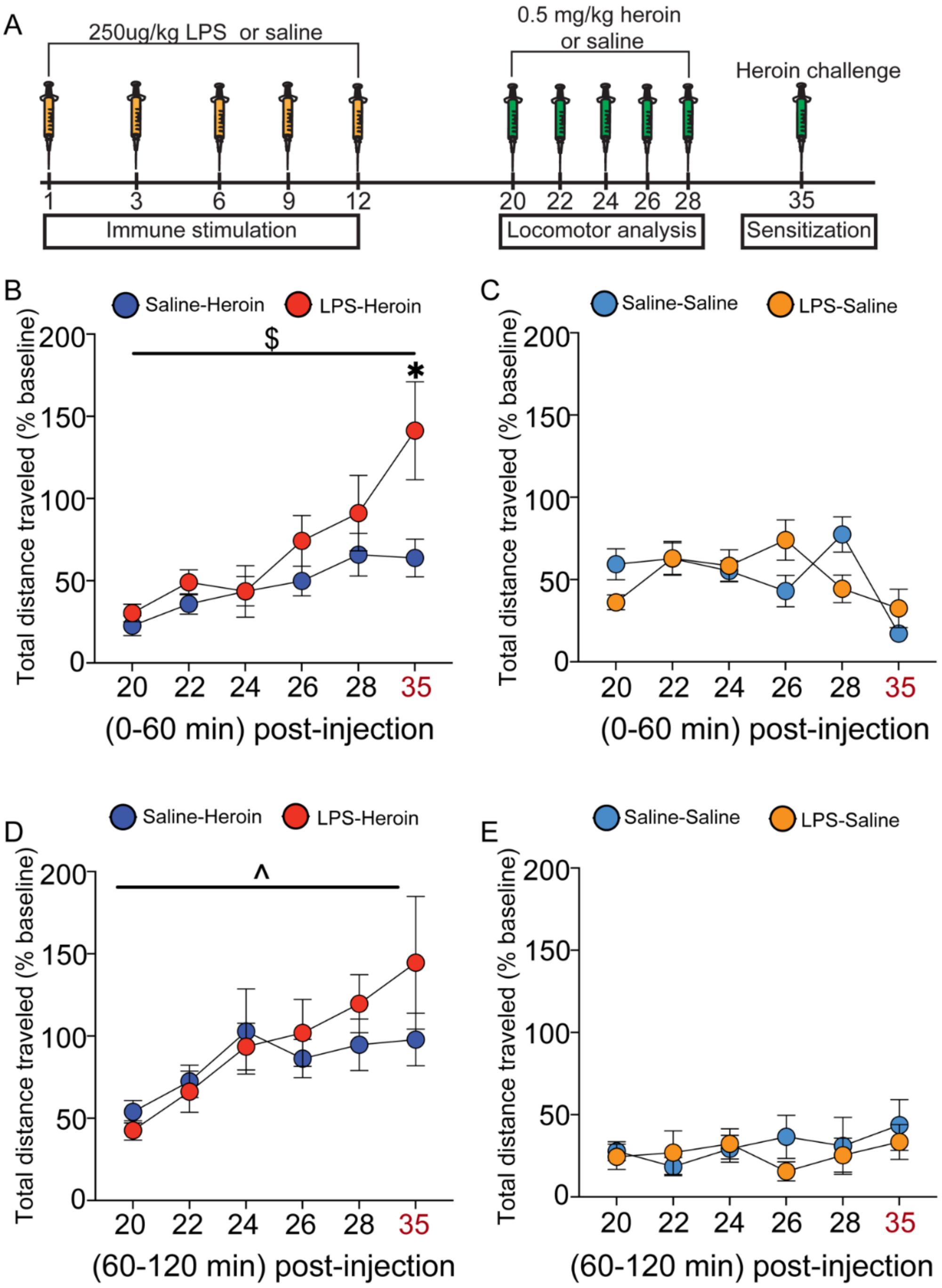

A
250ug/kg LPS or saline
0.5 mg/kg heroin or saline
Heroin challenge
1 3 6 9 12 20 22 24 26 28 35
Immune stimulation
Locomotor analysis
Sensitization
B
Saline-Heroin
LPS-Heroin
$
*
Total distance traveled (% baseline)
(0-60 min) post-injection
C
Saline-Saline
LPS-Saline
Total distance traveled (% baseline)
(0-60 min) post-injection
D
Saline-Heroin
LPS-Heroin
^
Total distance traveled (% baseline)
(60-120 min) post-injection
E
Saline-Saline
LPS-Saline
Total distance traveled (% baseline)
(60-120 min) post-injection

Supplemental Figure 7: Lever responses and infusions for animals that underwent heroin self-administration.

Animals underwent heroin or saline self-administration for 10 days. (A, B) The average number of active or inactive lever responses made over 10 days of self-administration for heroin (A) or saline (B) animals. (C) The average number of heroin or saline infusions made over 10 days of self-administration. Colored dots represent an individual animal's responses, with the assignment of a specific color for each animal. Error bars indicate mean +/- S.E.M. Symbols above line indicate Unpaired t-test (* p>0.05; *** p>0.001)

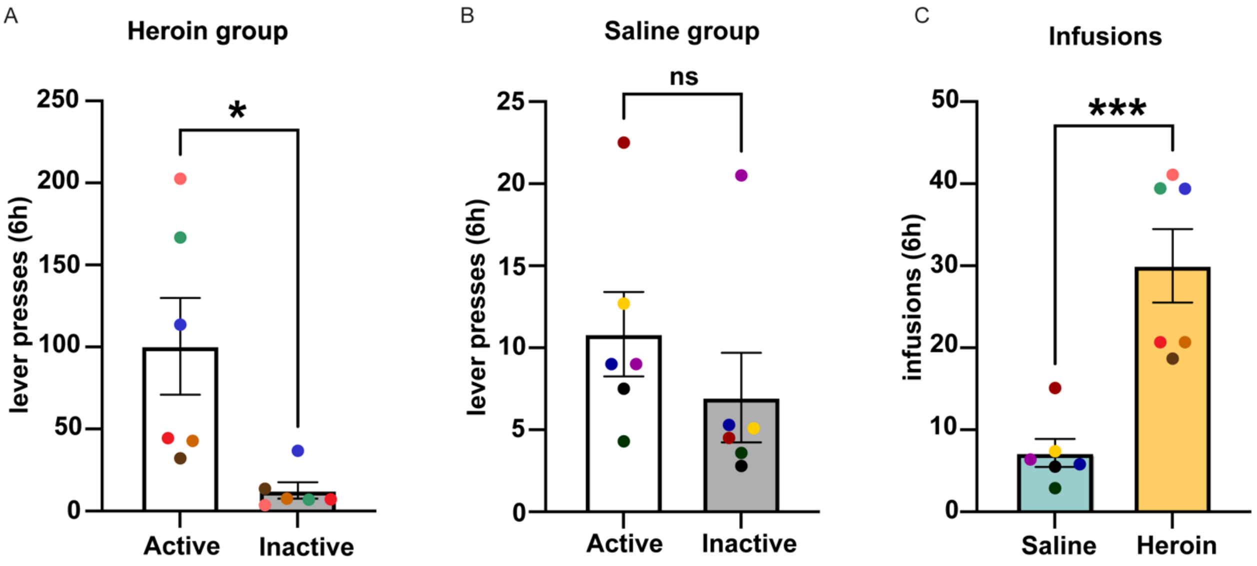

Supplemental Figure 8: Comparison of NAc transcriptome between Human OUD and Rat Heroin and/or LPS datasets. RRHO plots comparing the NAc transcriptome between the human OUD dataset from Seney et al and LPS-Saline (A); Saline-Heroin (B); LPS-Heroin (C); or Heroin-SA (D).

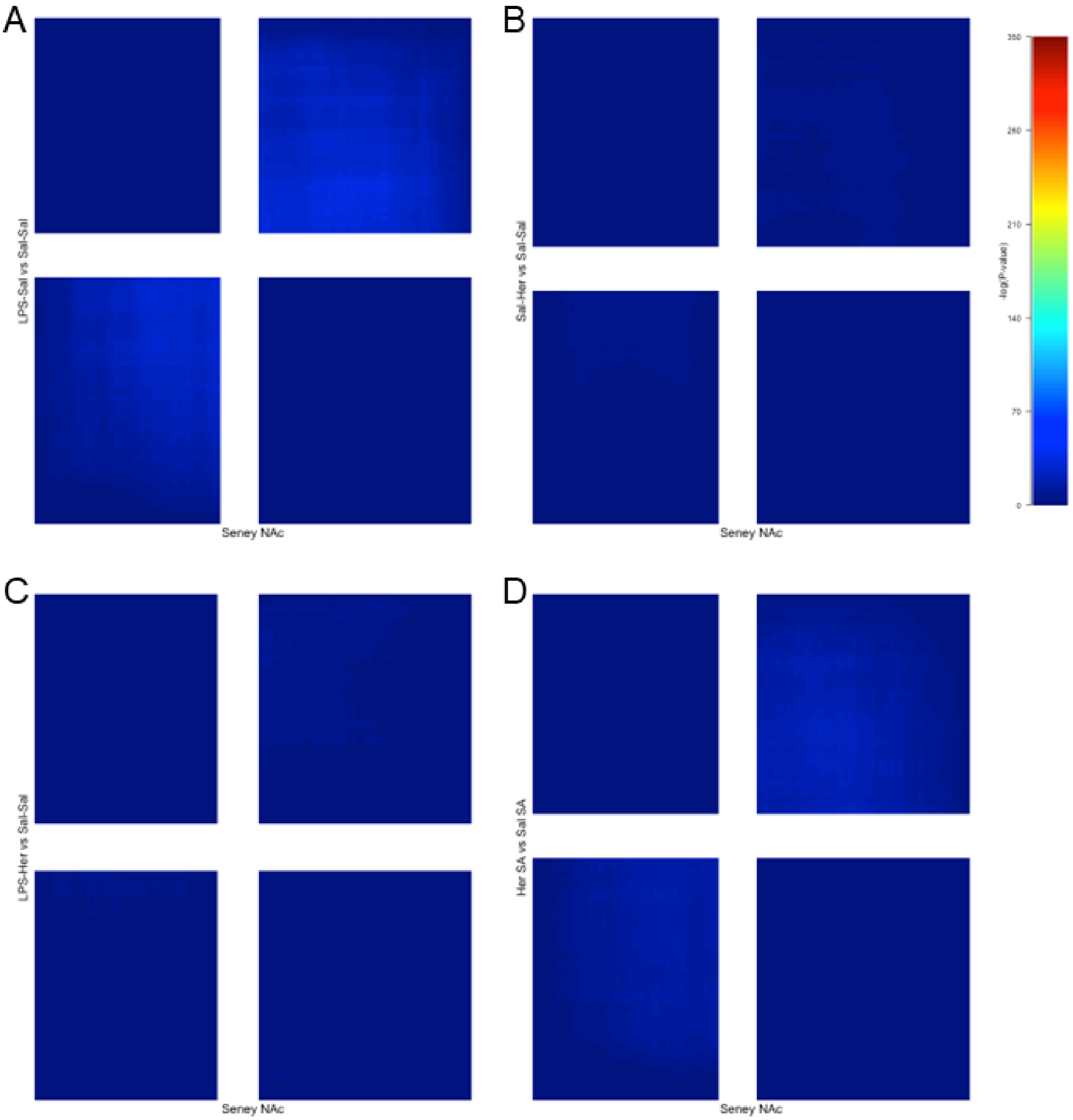

References:

Huang da, W., B. T. Sherman and R. A. Lempicki (2009). "Systematic and integrative analysis of large gene lists using DAVID bioinformatics resources." Nat Protoc **4**(1): 44-57.

Langmead, B., C. Wilks, V. Antonescu and R. Charles (2019). "Scaling read aligners to hundreds of threads on general-purpose processors." Bioinformatics **35**(3): 421-432.

Liao, Y., G. K. Smyth and W. Shi (2014). "featureCounts: an efficient general purpose program for assigning sequence reads to genomic features." Bioinformatics **30**(7): 923-930.

Love, M. I., W. Huber and S. Anders (2014). "Moderated estimation of fold change and dispersion for RNA-seq data with DESeq2." Genome Biol **15**(12): 550.

Seney, M. L., S. M. Kim, J. R. Glausier, M. A. Hildebrand, X. Xue, W. Zong, J. Wang, M. A. Shelton, B. N. Phan, C. Srinivasan, A. R. Pfenning, G. C. Tseng, D. A. Lewis, Z. Freyberg and R. W. Logan (2021). "Transcriptional Alterations in Dorsolateral Prefrontal Cortex and Nucleus Accumbens Implicate Neuroinflammation and Synaptic Remodeling in Opioid Use Disorder." Biol Psychiatry **90**(8): 550-562.
